# Clinical considerations of CDK4/6 inhibitors in HER2 positive breast cancer

**DOI:** 10.3389/fonc.2023.1322078

**Published:** 2024-01-16

**Authors:** Cui Zhang, Fulin Zhou, Jiali Zou, Yanman Fang, Yuncong Liu, Libo Li, Jing Hou, Guanghui Wang, Hua Wang, Xiaolian Lai, Lu Xie, Jia Jiang, Can Yang, Yisidan Huang, Yingji Chen, Hanqun Zhang, Yong Li

**Affiliations:** ^1^ Zunyi Medical University, Zunyi, China; ^2^ Maternal and Child Health Care Hospital of Guiyang City, Guiyang, China; ^3^ Department of Oncology, Guizhou Provincial People’s Hospital, Guiyang, China; ^4^ Department of Breast Surgery, Guizhou Provincial People’s Hospital, Guiyang, China; ^5^ Department of Digestive, People’s Hospital of Songtao Miao Autonomous County, Tongren, China; ^6^ Guizhou University of Traditional Chinese Medicine, Guiyang, China

**Keywords:** CDK4/6 inhibitor, HER2-positive breast cancer, off-label indications, abemaciclib, palbociclib, ribociclib

## Abstract

Deregulation of cell cycles can result in a variety of cancers, including breast cancer (BC). In fact, abnormal regulation of cell cycle pathways is often observed in breast cancer, leading to malignant cell proliferation. CDK4/6 inhibitors (CDK4/6i) can block the G1 cell cycle through the cyclin D-cyclin dependent kinase 4/6-inhibitor of CDK4-retinoblastoma (cyclinD-CDK4/6-INK4-RB) pathway, thus blocking the proliferation of invasive cells, showing great therapeutic potential to inhibit the spread of BC. So far, three FDA-approved drugs have been shown to be effective in the management of advanced hormone receptor positive (HR+) BC: palbociclib, abemaciclib, and ribociclib. The combination strategy of CDK4/6i and endocrine therapy (ET) has become the standard therapeutic regimen and is increasingly applied to advanced BC patients. The present study aims to clarify whether CDK4/6i can also achieve a certain therapeutic effect on Human epidermal growth factor receptor 2 positive (HER2+) BC. Studies of CDK4/6i are not limited to patients with estrogen receptor positive/human epidermal growth factor receptor 2 negative (ER+/HER2-) advanced BC, but have also expanded to other types of BC. Several pre-clinical and clinical trials have demonstrated the potential of CDK4/6i in treating HER2+ BC. Therefore, this review summarizes the current knowledge and recent findings on the use of CDK4/6i in this type of BC, and provides ideas for the discovery of new treatment modalities.

## 1 Introduction

Breast cancer (BC) is now a well-known type of cancer, accounting for 11.7% of all malignancies (1), and is the leading reason for cancer-associated death in women globally (2). At present, breast cancer is classified into five distinct subtypes based on genetic and epigenetic factors. These include luminal A, luminal B, HER2-positive, triple-negative A, and triple-negative B subtypes (3). Human epidermal growth factor receptor 2 positive (HER2+) is a molecular sub-type of BC that causes 15-20% of all BC cases (4). This type of BC is particularly aggressive, often with an uncertain prognosis and a high risk of disease recurrence (5, 6). HER2+ BC is defined as a molecular sub-type that has increased HER2 protein expression by immunohistochemistry (IHC) or has amplified HER2 gene expression by *in situ* hybridization (ISH). The following conditions can indicate HER2+: 1. The IHC result is IHC3+; 2. The IHC result is IHC2+, ISH dual-probe test results show that the HER2/chromosome enumeration probe 17 (CEP17) ratio is maintained at <2.0 and HER2 signal per cell is ≥6.0, or the HER2/CEP17 ratio is ≥2.0 and HER2 signal per cell is ≥4.0 (7).

Targeted therapies can alleviate HER2+ BC, mainly anti-HER2 antibodies such as trastuzumab and pertuzumab, and small molecule tyrosine kinase inhibitors (TKI), such as lapatinib and neratinib (8). The recommended treatment regimen for HER2+ metastatic breast cancer (MBC) is trastuzumab plus pertuzumab and a taxane as primary treatment and trastuzumab emtansine, an antibody-drug conjugate, as the secondary treatment for patients with progressive disease (9–11). Chemotherapy is another treatment option. In the United States, stage II and III HER2+ BC guidelines prescribe neoadjuvant/adjuvant chemotherapy regimen of doxorubicin/cyclophosphamide paclitaxel and docetaxel/carboplatin (12), however, systemic chemotherapy often brings many serious side effects. Despite significant advancements in HER2+ BC treatment over the past 20 years, some early BC patients still experience relapses (13, 14), and some HER2+ MBC patients experience primary or secondary resistance (15, 16). In the end, the majority of HER2+ MBC patients pass away from their illness (17, 18).

Recently, many studies have begun to turn attention to chemotherapy-free regimens that combine targeted therapies with cell cycle inhibitors. According to the past treatment history, cell cycle inhibitors are sensitive to estrogen receptor positive human epidermal growth factor receptor 2 negative (ER+/HER2-) BC. Cell cycle inhibitors are mainly used in this type of BC, and have achieved very good responses in clinical practice. There is also some interest in whether cell cycle inhibitors can be used in HER2+ BC. According to some preclinical and clinical studies, cell cycle inhibitors may be used to treat HER2+ BC in the future, and these results may offer new potential therapeutic approaches and strategies.

In this review, we briefly describe the mechanism of action of CDK4/6i and its current therapeutic efficacy against HER2+ BC. We present clinical trials related to this use that seek to broaden the use of CDK4/6i beyond treating advanced hormone receptor positive (HR+)/HER2- BC.

## 2 Mechanism of action of CDK4/6 inhibitors

Normal cells have elaborate regulatory mechanisms to ensure the orderly progress of each phase of the cell cycle. However, cell cycle disorders often lead to cancer development (19). Among them, the cyclin D-cyclin dependent kinase 4/6-inhibitor of CDK4-retinoblastoma (cyclinD-CDK4/6-INK4-RB) is an essential pathway for cancer cells to modulate G1 to S, which is important for many cancer types’ initiation, development, and survival (20, 21). When this important pathway is deregulated, cancer cell proliferation increases and leads to many types of cancer occurrence, especially BC (22, 23).

**Figure 1:**
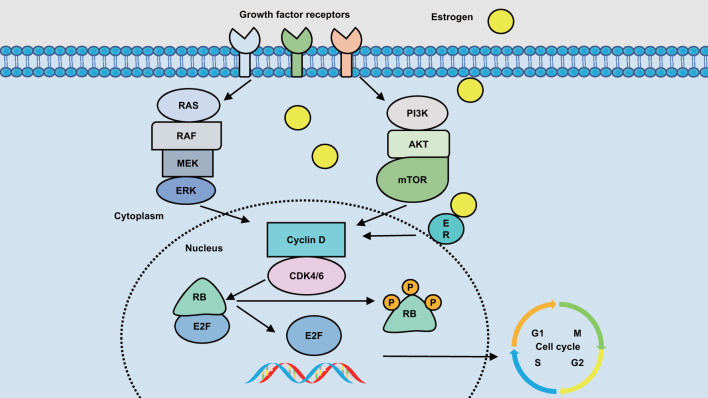
CDK4/6 a simple pathway to regulate G1 to S in cancer cells. Description: The transcription of D-type cyclins is influenced by various signaling pathways, including PI3K-AKT-mTOR, RAS-RAF-MEK-ERK, and ER. These pathways induce the expression and stability of D-type cyclins. CDK4/6 acts as a sensor that connects multiple signaling pathways to initiate and progress the cell cycle. CDK4 or CDK6 forms a complex with D-type cyclin, leading to the inactivation of the tumor suppressor Rb in the growth factor receptor pathway or estrogen receptor pathway. Consequently, the cell cycle transitions from the G1 phase to the S phase. Inhibitors of CDK4/6 can arrest the cell cycle in the G0/G1 phase by preventing downstream Rb phosphorylation through the inhibition of CDK4/6. ER estrogen receptor, RAS Ras proteins, RAF Raf kinase, MEK mitogentic effector kinase, ERK Extra cellular-signal-regulated kinases, PI3K phosphoinositide 3-kinase, Akt kinase, mTOR mammalian target of rapamycin, CDK cyclin-dependent kinase, RB retinoblastoma-associated protein, E2F protein.

In the cyclinD-CDK4/6-INK4-RB pathway, upstream signaling pathways, such as RAS and PI3K, promote the formation of cyclin D complexes with CDK4/6 by conveying external stimuli to cyclin D expression. This complex results in the phosphorylation of retinoblastoma (RB) protein, which inactivates RB (24). Inactivation of RB reduces RB’s repressive control of the E2F family of transcription factors. Inhibition of E2F transcription factors are reduced, E2F is dissociated from RB-E2F complex, and more E2F transcription factor is released (25). On one hand, the released E2F initiates DNA synthesis, leadingthe cell cycle from G1 to S. On the other hand, it promotes the transcription of E-type cyclin, activates CDK2, further phosphorylates RB1, reduces E2F inhibition, releases more E2F, promotes DNA synthesis (26), and forms a positive feedback loop (27). These mechanisms are shown in **Figure 1**.

As mentioned earlier, when the cell cycle is intact, it can be targeted by CDK4/6i, so CDK4/6i have become anti-tumor drugs. The FDA has highly acknowledged CDK4/6i, primarily abemaciclib, palbociclib, and ribociclib. When combined with targeted therapy, ET is the first choice of treatment for the majority of HR+/HER2- MBC patients (28). Although CDK4/6i are primarily used in HR+/HER2- BC, they also have potential use for other malignancies. For example, melanoma (29, 30), head and neck carcinoma (31, 32), esophageal carcinoma (33, 34), lung cancer (35), liver cancer (36), and other cancers reflect its extensive anti-tumor effect.

## 3 Clinical trials studying CDK4/6 inhibitors against HER2 positive breast cancer

Considering that extensive anti-tumor effects of CDK4/6i, especially the mechanism of action, cell cycle alternation in HER2+ BC (37), and the cyclinD/CDK 4/6 compound are directly downstream of the HER2 pathway (38), it is reasonable to apply CDK4/6i to HER2+ BC.

### 3.1 Preclinical trials studying

In some pre-clinical data, CDK4/6i treatment has been shown to remedy HER2+ BC. Nikolai et al. (2016) showed E2F1-driven DNA metabolism and replication of genes. Together with the phosphorylation and activity of the transcriptional coactivator steroid receptor coactivator-3 (SRC-3), E2F1-driven DNA metabolism is regulated by HER2 signaling to enhance BC cell proliferation. Furthermore, employing palbociclib, their analysis found a CDK signaling point that specifies the overlap and divergence of adjuvant pharmacologic targeting. Notably, E2F1 and its target genes are mainly disrupted by lapatinib and palbociclib, which tightly limit *de novo* DNA synthesis (39).

However, preliminary data from some early clinical trials indicate that only one CDK4/6i is ineffective against HER2-overexpressing BC, implying that combination therapy may be tried in HER2+ BC. Studies have found that the combination of small molecule inhibitors of HER2: TKI (e.g., pyrotinib, tucatinib, neratinib, etc.) and CDK4/6i appears to show some unexpected findings in preclinical studies of HER2+ BC. Zhang et al. (40) found that palbociclib improved the effects of pyrotinib in HER2+BC. The findings indicate that the therapeutic regimen of palbociclib and pyrotinib together is highly synergistic and has more antitumor activity than either drug alone. Together they cause a significant decrease in phosphorylated AKT (pAKT) and pHER3 activation, causing G0-G1 arrest, increasing apoptosis, and there is no appreciable increase in toxicity (40). Tucatinib combined with CDK4/6i also showed similar effects. The combined activity of tucatinib with the three approved CDK 4/6i, palbociclib, ribociclib, and abemaciclib, has been demonstrated in HER2+ BC. The combination increases sensitivity to cell inhibition compared to the single agents, while tucatinib and CDK4/6i have no antagonistic interactions, according to cell cycle research (41).

CDK4/6i has also shown a complementary mechanism of action to the dose-dependent effects of TKIs, in particular neratinib and afatinib. CDK4/6i inhibited proliferation/cell viability across multiple compounds in an additive relationship, which was summarized in different HER2 positive models (42). In addition, pre-clinical trials using neratinib and pabociclib in HER2 positive cell lines and patient-derived xenografts (PDX) confirmed the benefits of this combination. It is worth mentioning that the synergistic effect of the combination showed significantly enhanced anti-tumor efficacy, mainly in terms of tumor volume reduction (43). The combination of trastuzumab with abemaciclib also appears to show some therapeutic effect. In a HER2+ PDX model, no effect on xenograft growth was observed with trastuzumab alone, furthermore abemaciclib alone only inhibited tumor growth without causing regression. Remarkably, the combination of abemaciclib with trastuzumab led to both significant tumor cell growth inhibition and tumor regression (44).

### 3.2 Clinical trial studies

Several clinical trials studying the use of CDK4/6i and other drugs seem to confirm the safe and effective results observed in preclinical data. In the MonarcHER trial, 273 women with advanced ER+/HER2+ were enrolled and given a treatment combination of fulvestrant, abemaciclib, and trastuzumab, and then compared against standard chemotherapy plus trastuzumab. The study endpoint was reached after a median follow-up interval of 19.0 months. Results showed that the combination of cell proliferation inhibitors increased survival compared with standard chemotherapy, and that adverse reactions were tolerable (45). An NA-PHER2 study with multiple cohorts and multiple sites included 35 patients. The results showed a very interesting phenomenon in the combination of palbociclib with pertuzumab and trastuzumab. They found that Ki67 was lower after 2 weeks of treatment with this combination as well as at the time of surgery (6 weeks after treatment) compared with the beginning of the study (46). From the MonarcHER and NA-PHER2 studies, our hypothesis was that ER+/HER2+ individuals who do not want or cannot take chemotherapy could benefit from simultaneous inhibition of ER, HER2, and RB targets.

The SOLTI-1303 PATRICIA study compared palbociclib with trastuzumab, in combination with ET, to palbociclib with trastuzumab in highly pretreated patients with HER2+ advanced BC. These patients were also highly preconditioned, having received 2-4 lines of an anti-HER2 treatment. The results showed efficacy in this group of ER+/HER+ patients to be encouraging (47). Another phase 1b/2 study showed less consistent results. This combination treatment was safe but had limited resulting activity. The advanced HER2 + patients in this study had intensive pretreatment, including treatment with trastuzumab, pertuzumab, and trastuzumab emtansine. This indicates that patients who are too heavily pretreated in the metastatic setting and who then receive a median of 4 lines of chemotherapy, have a less than satisfactory response (48). These studies suggest that it is uncertain whether or not pretreatment is beneficial for advanced HER2+ BC patients who plan to use the CDK4/6i/anti-HER2 combination treatment. Given the relatively small population sizes in both studies, this may have contributed to some of the differences in results.

The aforementioned results indicate that CDK4/6i and other medications that are used together would provide extra therapeutic benefit for HER2 patients, regardless of pretreatment, therefore more research into this area is needed. Many new CDK4/6i and HER2-targeted medication combination schemes are now being investigated for treating both ER+/HER2+ and HR+/HER2+ breast cancer. These combinations are listed in **Tables 1**, **2**.

**Table 1:** Clinical trials studying the application of CDK4/6 inhibitors in HER2+ breast cancer.

Identifier	Study design	Agents and dose	Participants and recruitment period	Estimated/Actual enrollment	Primary endpoint and duration	Status
NCT03530696	single arm Open label Phase II	Palbociclib: 125mg T-DM1: 3.6 mg/kg	Metastatic HER2+BC and other breast tumors December 6, 2018- December 22, 2022	46	PFS 4 years	Completed No Results Posted
NCT03993964	single arm open label Phase II	Pyrotinib: 400mg SHR6390: 125mg	Metastatic Her2+BC August 15, 2019-October 30, 2020	20	ORR 100 months	Unknown No Results Posted
NCT04293276	single arm open label Phase II	Pyrotinib: ND SHR6390: ND	Metastatic Her2+BC April 1, 2020-August 23, 2021	41	ORR 2 years	Active, not recruiting NCT04293276
NCT03304080	single arm open label Phase I/II	Anastrozole:1 mg Palbociclib:100 mg/125mg Trastuzumab: 6 mg/kg or 8mg/kg Pertuzumab:420mg/840mg	Metastatic Her2+BC December 20, 2017- July 2024	44	DLT MTD CBR 3 months	Active, not recruiting No Results Posted
NCT03284723	Randomized Open Label Phase I	PF-06804103:ND Palbociclib : NDLetrozole : ND	Her2-/HER2+BC November 1, 2017- August 31, 2021	95	DLTs PFS TTP and DR 2 years	Completed Results SubmittedNotPosted
NCT05319873	Randomized Open label Phase Ib/II	Carboplatin : NDDocetaxel : ND Fulvestrant : ND Ribociclib : No Trastuzumab : ND Pertuzumab : ND Tucatinib : ND	Locally advanced/Metastatic Her2+BC and other breast tumors April 7, 2022- April 1, 2024	18	MTD、pCR 30 days or 58 days	Recruiting No Results Posted
NCT04095390	Randomized Open Label Phase II	Pyrotinib:400 mg SHR6390: 125mg Letrozole: 2.5mg Capecitabine: 500mg	prior trastuzumab-treated advanced HER2+BC September 30, 2019-November 30, 2021	60	ORR 2 months or 3 years	Unknown No Results Posted
NCT02657343 (48)	Non-randomized open label Phase I/II	Ribociclib:300/400/500/600mg T-DM1: ND Trastuzumab: 6 mg/kg Fulvestrant : ND	Advanced/Metastatic Her2+BC March 2016-March 2017 Median follow-up was 12.4months	13	RP2D:400mg CBR : NR mPFS:10.4months 10.9 months	Completed Has Results
NCT03054363 (49)	Single Group Open Label Phase Ib/II	Tucatinib:300mg Palbociclib: 75mg/125mg Letrozole:2.5mg	Metastatic Her2+BC November 2017- April 2020 The median follow-up was 33.6 months	42	Ib mPFS:8.2 months II mPFS:10.0months 4 years	Active, not recruiting Has Results
NCT04778982	parallel arm Open Label Phase II	KN026: 20 mg/kg Palbociclib: 125 mg Fulvestrant:500mg	Metastatic Her2+BC May 25, 2022- March 15, 2023	36	DLT、ORR 24 weeks or 1 year	Terminated No Results Posted
NCT02448420 (47)(PATRICIA II)	Randomized Open Label Phase II	Palbociclib: 125/200 mgTrastuzumab: 8mg/kg or 600mg Endocrine therapy Chemotherapy : NDAntibody-Drug Conjugates: 3.6 mg/kg	Previously-treated Locally Advanced or Metastatic Her2+BC July 2015 - November 2018 No median follow-up time	72	Cohort A:mPFS:4.2 months Cohort B1:mPFS: 6.0 months Cohort B2:mPFS: 5.1 months 6 months or 4 years	Active, not recruiting
NCT05429684	Non-randomized Open label Phase III	Trastuzumab: 6mg/kgPertuzumab:420mg Nab paclitaxel:200mg Pyrotinib:400mg Capecitabine T-DM1:3.6mg/kg Everolimus:4mg CDK4/6 inhibitor: Palbociclib:125mg AI: Letrozole 2.5mg Anti-PD-1monoclonal antibody:200mg	Advanced Her2+BC January 1, 2021- February 28, 2024	120	ORR、 PDO model inhibition rate six weeks or during the procedure	Recruiting No Results Posted
NCT03065387	Non-randomized Open label Phase II	Everolimus Neratinib : NDPalbociclib : ND Tra metinib:ND	Advanced Cancer Subjects With HER2 Mutation/Amplification and other type Mutation/Amplification October 31, 2017-October 1, 2025-	93	safety and tolerability 、MTD、DLT 28 days or 58 days	Active, not recruiting No Results Posted

BC, Breast Cancer, PFS, progression-free survival, ORR, Objective Response Rate, DLT, Dose-Limiting Toxicity, MTD, Maximum Tolerated Dose, CBR, Clinical Benefit Rate, DLTs, Dose-Limiting Toxicities, TTP, Time to Tumor Progression, DR, Duration of Response, RP2D, Recommended Phase2 Dose, pCR, Pathologic complete response, PDO, Patient-Derived Oranoid. ND, No Dose, NNR, Not reach.

**Table 2:** Clinical trials studying the application of CDK4/6 inhibitors in ER+/HER2+ or HR+/HER2+breast cancer.

Identifier	Study design	Agents and dose	Participants and recruitment period	Estimated/Actual enrollment	Primary endpoint and duration	Status
NCT02675231 (45)(monarcHER)	Randomized Open Label Phase II	(Abemaciclib)LY2835219:150mg Trastuzumab:8mg/kg Fulvestrant:500mg	Locally advanced/Metastatic HR+/Her2+BC May 31, 2016, and February 28, 2018 The median follow-up was 19.0 months	237	groupA mPFS:8.3months grougB mPFS:5.7months groupC mPFS:5.7months 36 Months	Active, not recruiting Has Results
NCT04224272	Non-randomized Open label Phase II	ZW25:ND Palbociclib : ND Fulvestrant : ND	HR+/Her2+BC June 10, 2020- April 28, 2023	51	DLT、Incidence of AEs 、PFS、 Incidence of lab abnormalities 4 weeks or 3.5 years or 6 months	Active, not recruiting No Results Posted
NCT03772353 (50) LORDSHIPS	single arm open label Phase Ib/II	Letrozole:2.5mg Pyrotinib:320mg Dalpiciclib(SHR6390):125mg Fulvestrant : ND	Advanced ER+/HER2+BC February 2019 - June 2020 The median follow-up was 11.4 months	15	ORR: 66.7% mPFS:11.3 months 1 year	Active, not recruiting No Results Posted
NCT02907918	Single arm Open label Phase II/III	Palbociclib:125mg Letrozole:25mg Trastuzumab:2mg/kg or 4mg/kg Goserelin:3.6mg	ER+/HER2+ BC June 30, 2017- August 24,2020	26	Number of Participants With pCR:2 pCR rate:8% 16 weeks	Terminated Has Results
NCT04858516	Single Group Open Label Phase II	Palbociclib : NDExemestane : NDTrastuzumab : NDPyrotinib : ND	ER+/HER2+BC April 30, 2021- April 30, 2024	57	pCR 24 weeks	Not yet recruiting No Results Posted
NCT03709082	Non-Randomized Open Label Phase I/II	Palbociclib:75/mg Letrozole:2.5mg T-DM1:3.6mg/kg	ER+/HER2+ Metastatic BC October 15, 2018- March 12, 2020	3	ORR 5 years	Active, not recruiting No Results Posted
NCT03644186 (51)	Randomized Open Label Phase II	Paclitaxel:80mg/m2 Trastuzumab:600mg Pertuzumab:840mg Palbociclib:125mg Letrozole:2.5mg	ER+/HER2+ Early BC April 16, 2019- January 3, 2023 No median follow-up time	144	No pCR No mPFS 16 weeks.	Completed No Results Posted
NCT05076695	Single Group Open Label Phase II	Palbociclib:125mg fulvestrant:500mg trastuzumab: 6mg/kg or 8mg/kg pyrotinib: 400mg	ER+/HER2+ BC October 15, 2021- October 15, 2023	37	pCR 1 year	Recruiting No Results Posted
NCT02947685 (52)(PATINA)	Randomized Open Label Phase III	Palbociclib:125mg trastuzumab: 6mg/kg or 8mg/kgpertuzumab:420mg or 840mg letrozole:2.5mg Anastrozole:1mg Fulvestrant:250mg Exemestane:25mg	HR+/HER2+ Metastatic BC June 21, 2017- December 30, 2023 No median follow-up time	496	No PFS 24 months	Active, not recruiting No Results Posted
NCT03913234	Single Group Open Label Phase I b/II	Ribociclib:200-600mg Trastuzumab:8mg/kg loading followed by 6mg/kg Letrozole:2.5mg	HR+/HER2+ Advanced BC Actual Study Start Date : June 10, 2019– October 30, 2023	95	PFS 1 year	Recruiting No Results Posted
NCT02530424 (46) (NA-PHER2)	Single Arm Open Label Phase II	Trastuzumab: 6mg/kg or 8mg/kg Pertuzumab:840mg Palbociclib:125mg Fulvestrant500mg	ER+/HER2+ BC May 20, 2015, -February 8, 2016 No median follow-up time	102	Serial measures of Ki67- At baseline Ki67:31·9 week 2:4·3 surgery:12.1 26 weeks	Completed No Results Posted

BC, Breast Cancer, PFS, progression-free survival, ORR, Objective Response Rate, DLT; Dose-Limiting Toxicity, pCR, Pathologic complete response, AEs, Adverse Events, AE, Adverse Event. ND, No Dose, NNR, Not reach.

## 4 Patients with HER2+ brain metastasis

Brain metastases (BM) are a common complication for many cancer patients, particularly for those with HER2+ BC (53). These individuals have a higher risk for developing BM (54), with an incidence of about 50%, increasing year by year (55). Patients with this type of breast cancer often have a poor quality of life and poor survival chances (56). Current treatments for such patients include radiotherapy, surgery, and HER2 targeted therapy. Radiotherapy is the main treatment for BM, but it is often associated with neurocognitive decline and has an unclear prognosis (57). HER2 targeted medicines are unable to pass the blood-brain barrier (BBB). Reliable information on how to handle HER2+ BM is lacking. Despite international consensus guidelines recommending a sequential HER2 blockade, it is unclear which anti-HER2 agent is the best choice when BC occurs (58). Therefore, it is necessary to find a systemic therapy that may effectively cross the BBB and avoid the neurocognitive decline caused by radiation therapy.

In some studies, a series of new and highly effective CDK4/6i have been designed and synthesized, which show good BBB permeability in the therapies treating glioblastoma multiforme (59). In contrast, CDK4 and CDK6 inhibitors have been shown to reach high brain concentrations in rodents in preclinical studies and demonstrate the advantages of abemaciclib, which may require lower doses and longer durations than palbociclib (60). Ni et al. (2022) and his colleagues found that combination therapy with tucatinib and abemaciclib could reduce tumor growth and significantly and prolong survival time in mouse models of HER2+ BC with brain metastases, while tucatinib or abemaciclib as monotherapy did not show significant therapeutic benefit (61). Therefore, the use of CDK/6i, either alone or together, may be a potential therapy option for individuals with BM.

The primary goal of the phase II clinical trial NCT02774681 in HER2+ BM was to determine whether palbociclib is effective in HER2+BC patients with BM. In this study, a total of 12 patients were enrolled in a daily oral palbociclib regimen, repeated every 28 days. NCT04334330 is a non-randomized, phase II clinical trial in ER+/progesterone receptor-positive (PR+)/HER2+ BC with BM. This study’s main objective was to evaluate the effectiveness of palbociclib, trastuzumab, and pyrotinib in combination with fulvestrant in ER+/PR+/HER2+ BC with BM. The regimen is daily oral palbociclib on days 1 to 21, with intravenous trastuzumab every three weeks, daily oral pyrotinib, and intramuscular fulvestrant every 4 weeks. Cycles were repeated every 28 days. As shown in **Table 3**.

**Table 3:** Clinical trials studying the application of CDK4/6 inhibitors in Patients with HER2+ brain metastasis.

Selected inclusion/Exclusion Criteria	Interventions	Primary End point
**NCT04334330 (** 62) **A Phase II study to Evaluate the Efficacy of Palbociclib, Trastuzumab and Pyrotinib With Fulvestrant in ER/PR+ and HER2+ breast cancer patients with brain metastasis**		Status : Recruiting
**Estimated Enrollment: 34** - Histologically confirmed ER/PR positive, HER2-positive metastatic breast cancer - Measurable disease in the brain, defined as at least 1 lesion measuring >= 10 mm on MRI at the time of registration - leptomeningeal disease or been treated with WBRT is not allowed	- palbociclib PO daily on days 1-21, combined with trastuzumab IV every three weeks, pyrotinib PO daily and fulvestrant IM every 4 weeks. Cycles repeat every 28 days - No specific drug dosage - Actual Study Start Date : December 4, 2020 Estimated Primary Completion Date : December 30, 2023	Current results: Objective response rate in the CNS: 28.6%, mPFS:10.6 months, The time to progression in the CNS was 8.5 months The median follow-up was 6.3 months duration:3 years
**NCT02774681** **A Phase II Single Arm Study to evaluate the Efficacy of Palbociclib in Patients With Metastatic HER2-positive Breast Cancer With Brain Metastasis**		Status: Terminated Has Results
E**stimated Enrollment: 12** - Histologically confirmed HER2-positive metastatic breast cancer - should not have received > 2 lines of chemotherapy for metastatic disease - Measurable disease in the brain, defined as at least 1 lesion measuring >= 5 mm on imaging at the time of registration - Any uncontrolled neurological symptom attributed to CNS metastasis or leptomeningeal disease or Previous treatment with Palbociclib is not allowed	- palbociclib PO daily on days 1-21. Courses repeat every 28 days in the absence of disease progression or unacceptable toxicity - trastuzumab IV over 30-90 minutes every 3 weeks - No specific drug dosage - Recruitment period: May 25, 2016- January 28, 2019	No RRR, Stable DiseaseCNS:6, Progressive Disease CNS:6 duration:3 years

RRR, Radiographic Response Rate in the CNS in Patients With HER2-positive Breast Cancer Who Have Brain Metastasis Treated With Palbociclib. No RRR was not calculated as the study did not met statistical analysis criteria due to study closing before total accrual was met.

## 5 CDK4/6 inhibitors overcome resistance to targeted therapy in HER2 positive breast cancer

**Figure 2:**
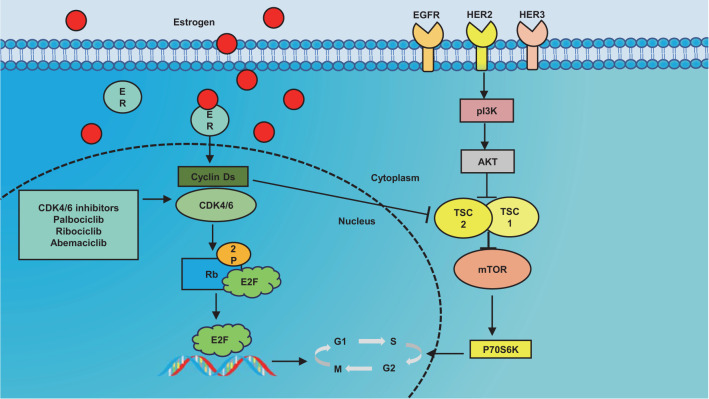
Simple association diagram between human epithelial growth factor receptor 2 (HER2) and estrogen receptor (ER) pathways. Description: Estrogen has the ability to enhance cell proliferation by increasing the levels of cyclin D1, CDK4/6 activity, and cyclin E/CDK2 levels. Additionally, HER2 can influence the PI 3 K/Akt/mTOR pathway to regulate cell proliferation. It is worth noting that these two pathways can be interconnected through TSC2. This implies that the D-CDK 4/6 pathway plays a crucial role in inhibiting the HER 2 pathway and serves as a fundamental principle for overcoming resistance to HER2 inhibitors. EGFR epidermal growth factor receptor, ER estrogen receptor, HER Human epidermal growth factor receptor, PI3K phosphoinositide 3-kinase, Akt kinase, mTOR mammalian target of rapamycin, CDK cyclin-dependent kinase, RB retinoblastoma-associated protein, E2F protein, p70S6K is members of the serine/threonine protein kinase family, TSC1 proteins and TSC2 proteins.

The use of HER2 inhibitors, especially in combination, provides significant therapeutic benefits to BC patients, but the response is often limited due to persistent primary or acquired resistance (63–65). There are currently numerous hypothesized pathways for trastuzumab resistance in BC that is HER2 positive. The primary signaling pathways that HER2 mediates are the RAS/MAPK, PI3K/PKB/Akt, and IL6/JAK/STAT3 pathways. These pathways are crucial for cell growth, differentiation, skeleton construction, cell death, and malignant transformation (66, 67). Among them, PI3K/Akt/mTOR pathway and cyclin D1/CDK4/6/retinoblastoma protein (pRb) axis are important resistance pathways for HER2 targeted therapy (68), as shown in **Figure 2**.

Over-activation of the PI3K/AKT/mTOR pathway is thought to be among the dominating causes of carcinogenicity, which is linked to various resistance mechanisms to anti-HER2 therapy (69). Pre-clinical evidence has shown that the PI3K/Akt/mTOR pathway contributes to HER2-directed therapy resistance, making it a new target for the treatment of HER2-resistant disease in clinical development (70). This has sparked a number of trials to test whether or not inhibitors of this pathway can overcome HER2-directed therapy resistance. Even though mTOR inhibitors were the main focus of the majority of these trials, they produced encouraging outcomes (71).

Downstream from the HER2 signaling pathway, the Cyclin D-CDK4/6 pathway is important in blocking the HER2 pathway (38). In actuality, HER2 targeted therapy-resistant recurrent tumor cells are susceptible to RNA interference or CDK4/6 inhibitor-mediated cyclin D1 down-regulation (72). When cyclin D1 is activated downstream, trastuzumab and other HER2 targeted medicines become resistant to their effects.

Studies indicate that CDK4/6i and HER2 inhibitors used together yield some intriguing results in the subsequent treatment of HER2+ BC. Goel et al. (2016) used cell line-based mechanistic investigations and clinical transgenic mouse models to discover that CDK4/6i can inhibit RB phosphorylation and decrease tuberin (TSC2) phosphorylation, thereby inhibiting mTORC1/S6K/S6RP activity. Dual inhibition of epidermal growth factor receptor (EGFR)/HER2 and CDK4/6 can more effectively enhance this effect, which relieves feedback inhibition of upstream EGFR family kinases and resensitizes tumors to EGFR/HER2 blockade. In transgenic mouse models, HER2 and CDK4/6i collaborated to inhibit cell proliferation, control tumor growth *in vivo*, and delay tumor recurrence (44). Another study of Qingfei Wang and his colleagues showed less consistent results. In transgenic mouse models, the combination of anti-HER2 and CDK4/6i rapidly developed resistance. Two weeks of continuous anti-HER2/neu antibody plus palbociclib produced significant results: tumor regression, 52.74% average volume reduction, and significant inhibition of tumor cell proliferation, efficacy, and prolonged survival. Tumors treated with this combination, however, rebounded and eventually developed resistance shortly after tumor regression. However, they discovered that switching to a combination immunotherapy containing Cabo, a potential MDSC/IMCs targeting inhibitor, could overcome resistance to the anti-HER2/neu antibody plus palbociclib (73).

In fact, these findings not only generated interest in the use of CDK4/6i in HER2+BC therapy, but they also demonstrated that the simultaneous treatment of HER2 targeted drugs and CDK4/6i is effective, and these two inhibitors may work well in combination.

## 6 Predictive biomarkers of sensitivity to CDK4/6 inhibitors

CDK4/6i combined with ET is the main therapeutic strategy for HR+/HER2- BC patients with metastasis. However, resistance to CDK4/6i leads to treatment failure and cancer progression. Treatment strategies for reducing CDK4/6 resistance have not yet been standardized, and reliable biomarkers of treatment response need to be identified, particularly in persons with HER2+ BC.

Raspé et al. (2017) found that CDK4 T172 phosphorylation was most closely connected to breast tumors cell line susceptibility to the particular CDK4/6 inhibitor PD0332991 (palbociclib). The primary rate-limiting step for CDK4 activation is CDK172-activated T4 phosphorylation, which binds cyclin D. In the study, gene expression profiles identified tumors that were less responsive to CDK4/6i. This response suggests that sub-population sensitivity studies to this agent may help guide its use in cases of HER2+ and basal-like tumors (74).

It was found that HER2-E tumor cells were sensitive to anti-HER2 therapy but did not die and acquired the luminal A phenotype. This is particularly important in HR+/HER2+ disease. This phenotype develops relatively quickly and leads to anti-HER2 resistance. Surprisingly, after exposure to the anti-HER2 pathway, palbociclib in combination with anti-HER2 therapy has been shown to be more effective. These results demonstrate the luminal A phenotype can serve as a biomarker of anti-HER2 remedy resistance and implies that developing a more lumen-like phenotype may make cells more susceptible to CDK4/6i. It’s interesting to note that the HER2 targeted remedy boosted sensitivity to CDK4/6i by enhancing the luminal phenotype. Finally, discontinuing the *in vitro* HER2 targeted remedy or developing resistance to the anti-HER2 remedy causes the original HER2-E phenotype to return. Our findings encourage the development of treatment strategies using the CDK4/6i sub-type switching and maintaining the anti-HER2 remedy (75).

The findings of a different study point to the potential use of pRb as a biomarker to forecast CDK4/6i responsiveness in HER2+BC. A correlation between the number of HER2 gene copies and pRb levels was observed in the 77 HER2+ cases that were investigated. This data suggests that the number of copies of the HER2 gene can be used to predict CDK4/6 activity, with more copies indicating higher CDK4/6 activity. In order to discover the best course of treatment, it might be necessary to take into account the drug dose related to the number of HER2 gene copies, if CDK4/6i is ever to be considered for a remedy for HER2+ BC (76).

## 7 Conclusions

HR+ BC has responded well to ET in combination with CDK4/6i. Still, research continues to search for more treatments. Preclinical research has been done on xenografts and HER2+ BC cell lines using CDK4/6i. Simultaneous targeting of HER2 and CDK, or DNA replication may be a suitable approach, but more clinical trials with larger sample sizes are essential for evaluating the benefits and drawbacks of CDK4/6i-based treatment regimens. At present, there are many effective targeted drugs for HER2+ BC, but their drug resistance often limits their clinical use.

Combining CDK4/6i with anti-HER2 therapy, such as trastuzumab and patuzumab, along with small-molecule tyrosine kinase inhibitors, has shown promise as a treatment modality. This regimen has demonstrated a higher survival benefit, with manageable adverse effects. Additionally, the combination of CDK4/6i and anti-HER2 targeting has been found to overcome anti-HER2 resistance, synergistically inhibiting cell proliferation, controlling tumor growth *in vivo*, and delaying tumor recurrence. However, it should be noted that this combination therapy can eventually lead to drug resistance. Nevertheless, studies suggest that combining it with certain immunotherapies may help overcome this resistance. Surprisingly, the CDK4/6i combined with anti-HER2 treatment has also shown good efficacy in treating HER2+ BM in BC. Therefore, for HER2+ BC patients who are unable or unwilling to undergo chemotherapy, the combination of CDK4/6i and anti-HER2 treatment could be a potential option, offering hope for extended survival.

In addition, if this combination therapy is a worthwhile option, more thorough clinicopathological characteristics and biomarkers of HER2+ BC sensitivity to CDK4/6i merit further investigation in pre-clinical research. And some clinical studies have even demonstrated the efficacy and safety of a three-drug regimen of CDK4/6i combined with endocrine therapy and anti-Her2 in HER2+ BC, an interesting chemotherapy-free combination. Our goal is to make better use of these novel targeted medications in the near future and give breast cancer patients more accurate and tailored care. Currently we are eagerly awaiting the outcomes of several trials of new CDK4/6i combinations.

## Author contributions

CZ: Writing – original draft. FZ: Writing – original draft. JZ: Writing – original draft. YF: Writing – original draft. YCL: Writing – original draft. LL: Writing – original draft. JH: Writing – review & editing. GW: Writing – review & editing. HW: Writing – review & editing. XL: Writing – original draft. LX: Writing – original draft. JJ: Writing – original draft. CY: Writing – original draft. YH: Writing – original draft. YC: Writing – original draft. HZ: Writing – review & editing. YL: Writing – review & editing.
